# Profibrogenic effect of high-mobility group box protein-1 in human dermal fibroblasts and its excess in keloid tissues

**DOI:** 10.1038/s41598-018-26501-6

**Published:** 2018-05-30

**Authors:** Won Jai Lee, Seung Yong Song, Hyun Roh, Hyo Min Ahn, Youjin Na, Jihee Kim, Ju Hee Lee, Chae Ok Yun

**Affiliations:** 1 Institute for Human Tissue Restoration, Department of Plastic & Reconstructive Surgery, Yonsei University College of Medicine, Seoul, Korea; 2 Department of Bioengineering, College of Engineering, Hanyang University, Seoul, Korea; 3 Department of Dermatology and Cutaneous Biology Research Institute, Yonsei University College of Medicine, Seoul, Korea

## Abstract

High-mobility group box 1 (HMGB1) protein acts as a DNA chaperone for nuclear homeostasis. It translocates into the cytosol and is secreted into extracellular spaces, triggering proinflammatory cytokines and acting as a mediator in fibrosis. We determined whether HMGB1 plays a role in normal dermal fibrosis and keloid, and is involved with transforming growth factor β. We investigated the translocation and active release of HMGB1 from normal dermal fibroblasts under lipopolysaccharide stimuli, and the redistribution of nuclear HMGB1 into the cytoplasm of keloid fibroblasts. HMGB1 and its effector toll-like receptors and receptors for advanced glycation end product proteins are actively expressed in keloid tissues. Exogenous HMGB1 can induce the proliferation of human dermal fibroblasts, and could act as a profibrogenic molecule to produce collagen, decrease MMP-1, and increase TIMP-1 mRNA expression. Moreover, administration of HMGB1 increased the expression level of TGF-β1 and internal signaling molecules, such as Smad 2 and 3, phosphorylated Smad 2/3 complex, Erk 1/2, Akt, and NF-κB. Collectively, we demonstrate that HMGB1 treatment increases the expression level of collagen types I and III, elastin, and fibronectin in dermal spheroid cultures, thus making HMGB1 a promising therapeutic target for treatment of profibrogenic diseases.

## Introduction

Keloid is a locally aggressive fibro-proliferative disorder of the skin. It is frequently accompanied by discomfort, contracture, and disfigurement^1^. Although a treatment for keloid is in great demand, there has been no definite therapeutic option because the mechanism underlying keloid development has not been fully elucidated^2–4^. Multiple causes have been proposed, including genetic, environmental, molecular signaling, and anatomical factors^1,4–6^. Inflammation is a well-known cause of keloid, and many researchers have assumed that it results from prolonged inflammation in localized areas^2,4,7^. The continuous infiltration of immune cells during prolonged and intense inflammation contributes to the continuous growth of keloid lesions^6,8^. Moreover, keloid involves an abnormal response to inflammation^9^.

Damage-associated molecular pattern molecules (DAMPs), including high-mobility group box 1 (HMGB1), heat shock proteins, purine metabolites, hyaluronan, heparin sulfate, IL-1α, and IL-33 initiate inflammatory responses^10,11^. HMGB1 is a nuclear, non-histone DNA-binding protein that acts as a DNA chaperone for nuclear homeostasis^12,13^. During cellular stress, it is released into the cytosol, and can exit the cell during loss of membrane integrity or active secretion^13,14^. In the cytoplasm, HMGB1 regulates cellular processes such as autophagy and apoptosis^15–17^. Extracellular HMGB1 interacts with several receptors and coordinates cellular responses of immune system activation, cell migration, cell growth, and tissue repair and regeneration. Extracellular HMGB1 also acts as a chemo-attractant factor that mediates the migration of monocytes and neutrophils. HMGB1 binds to receptors such as toll-like receptors (TLRs) and the receptor for advanced glycation end products (RAGE) to activate proinflammatory responses. Downstream signaling mediated by HMGB1 interaction involves mitogen-activated protein kinases (MAPKs) and nuclear factor kappa B (NF-κB), and facilitates cellular responses including cell migration and pro-inflammatory cytokine release^11,13,18–20^. Recently, release of HMGB1 following tissue injury is associated with fibrotic processes in systemic sclerosis^21^, liver fibrosis^13,22–24^, renal fibrosis^13,25^, and pulmonary fibrosis^21,26^, whereas HMGB1-related inhibitions provide protection against fibrosis. Therefore, HMGB1 may be an important mediator in fibrosis, and may be the key to a successful therapeutic strategy its treatment. However, there are only few reports about regarding its role in skin fibrosis, especially in keloid.

Because HMGB1 is a potent initiator of inflammation, and is associated with fibrogenesis in other disease models, and that HMGB1 and TGF-β1 may play similar roles in the induction of keloid development. In this study, we investigated the role of HMGB1 in skin fibrosis and keloid scar formation and its underlying mechanism. Our experiments elucidate the functions of HMGB1 and its crucial role in abnormal scarring process, and may lead to the acceptance of HMGB1 as a promising target for fibrotic disease therapy.

## Results

### Differential location of HMGB1 expression in normal human dermal fibroblasts (HDFs) and keloid fibroblasts (KFs)

**Figure 1:**
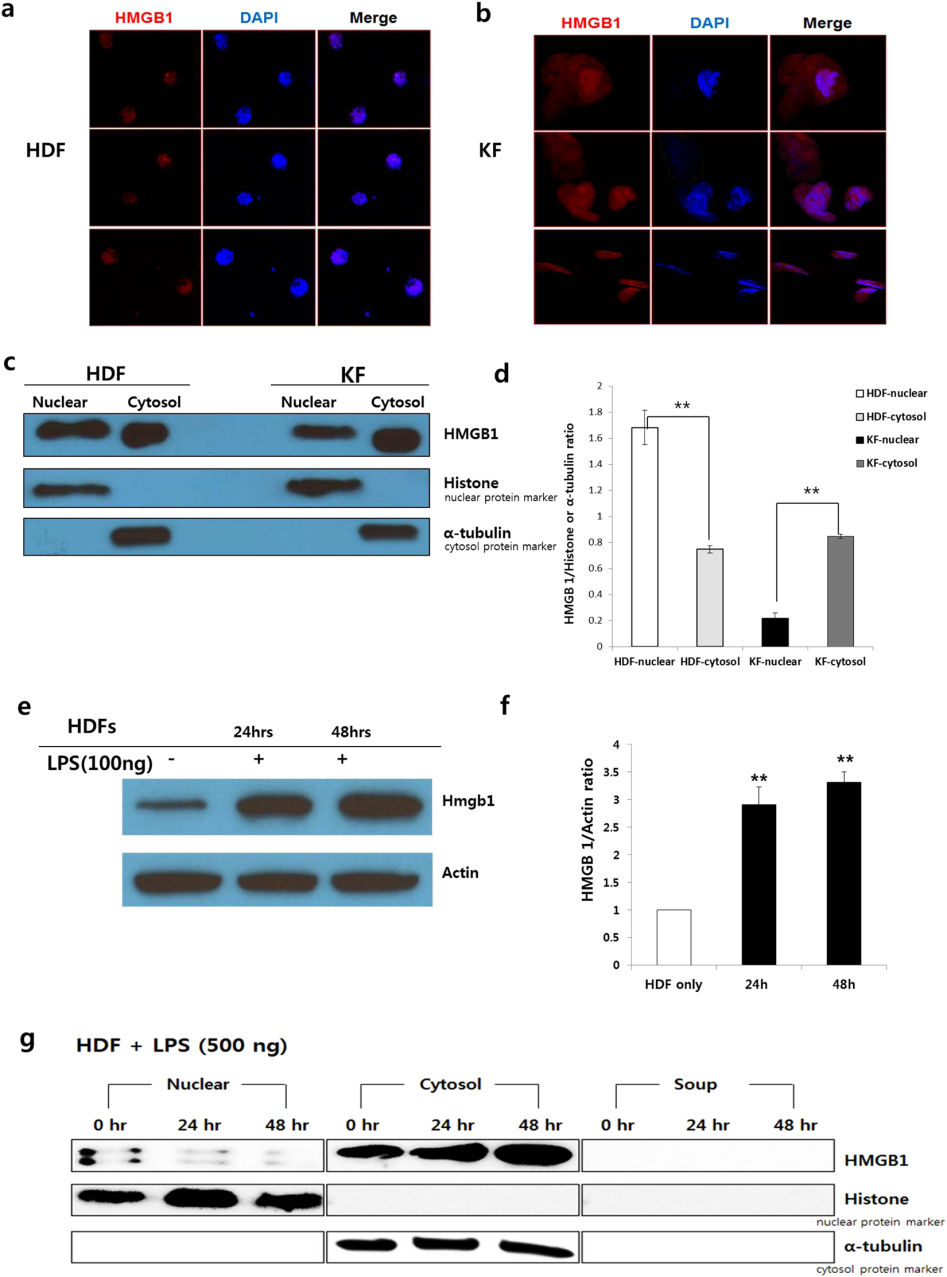
Differential location of HMGB1 expression in HDFs and KFs. (**a**) HMGB1 expression levels by immunofluorescence. HMGB1 expression (red) was mainly detected in the HDF nuclei. (**b**) HMGB1 expression was observed in KFs, but was more abundant in the cytosol and extracellular space. (**c** and **d**) HMGB1 protein expression. The levels of proteins were assessed by western blot. α-tubulin was used as a loading control for cytosol; histone was as a nuclei protein control. Relative levels of HMGB1 in the cytoplasmic fraction were significantly elevated in the KFs. (**e** and **f**) Stimulation of HDFs with LPS (100 ng/mL) for 24 h and 48 h resulted in increased time-dependent expression of HMGB1 protein in the cell lysate (**p < 0.01). (**g**) Whole cell lysates were fractionated and the levels of HMGB1 in the cytoplasmic and nuclear fractions were determined by western blotting to determine whether LPS treatment induced HMGB1 translocation. HMGB1 translocated from nucleus to cytoplasm in HDFs under LPS treatment (500 ng). Dermal fibroblasts cell line was purchased as primary cell line from the ATCC (American Type Culture Collection, Manassas, VA, USA). Primary keloid fibroblast cell line were obtained from human keloid explant under IRB protocol. Both cells were used for experiment after 2~3 passage.

Normally, HMGB1 shows predominantly nuclear localization, and is rarely exported from dermal fibroblasts. To evaluate whether HDF of KFs secrete HMGB1 during the external stress, main sites of HMGB1 expression is detected by immunofluorescence staining. HMGB1 expression (red) is mostly detected in the nuclei of HDFs (Fig. 1a). However, it was more abundant in the cytosol and/or extracellular space of KFs (Fig. 1b). Similar data were also obtained by western blotting analysis (Fig. 1c). HMGB1 protein is localized in the cytoplasm of KFs, but its distribution is mostly in the nucleus of HDFs (Fig. 1d).

Next, we evaluated whether lipopolysaccharide (LPS) stimulation induces the expression of HMGB1 protein in HDFs. As shown in Fig. 1e,f, the immunoblotting data demonstrate that stimulation of HDFs by LPS (100 ng/mL) for 24 h or 48 h resulted in an increase in the time-dependent expression of HMGB1 protein in the cell lysate (**p < 0.01). We subjected HDFs to cell fractionation, isolation of nuclear and cytoplasmic fractions, and western blotting analysis to determine whether nuclear–cytoplasmic translocation occurred during stimulation by an inflammatory mediator such as LPS (500 ng). As shown in Fig. 1g, the HMGB1 protein expression levels in the nuclear fractions were significantly reduced after 24 and 48 h of LPS treatment, but there was a significant increase in cytoplasmic HMGB1. From our results nuclear HMGB1 can translocate from the nucleus to the cytosol in the HDFs when stimulated by external LPS. Moreover, in KFs, the expression of HMGB1 was modified and relocalized to the cytosol or extracellular spaces.

### HMGB1 acts as a profibrotic molecule in HDFs

**Figure 2:**
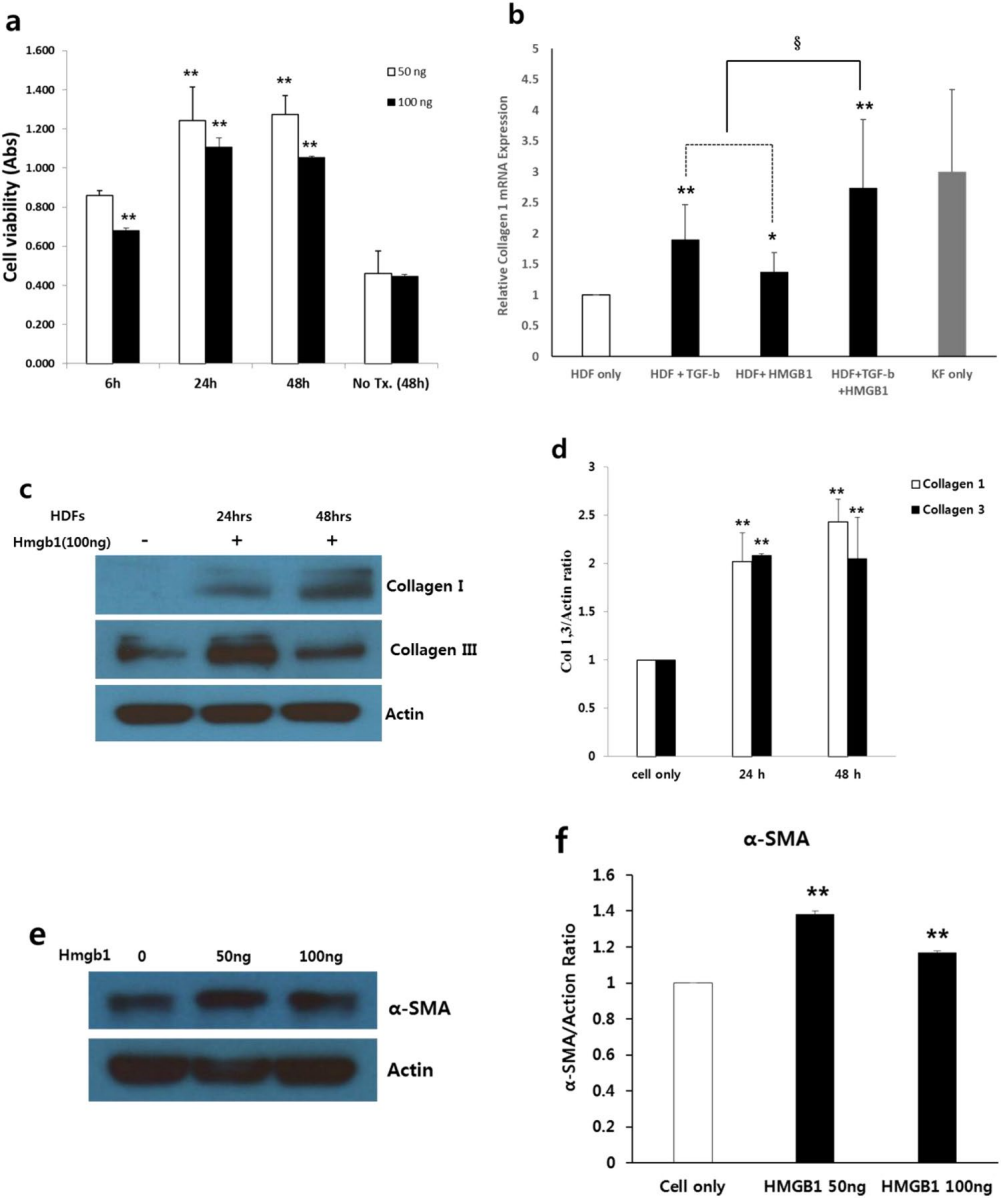
Role of HMGB1 as a profibrotic molecule. (**a**) An MTT assay revealed a significant increase in time-dependent proliferation activity in the HMGB1-treated HDFs (50 and 100 ng; **p < 0.01). (**b**) Type I collagen mRNA expression was equivalently increased in the HDFs treated with HMGB1 (100 ng) or TGF-β1 (10 ng) (*p < 0.05, **p < 0.01). There was also an additive increase of type I collagen mRNA expression after simultaneous treatment with HMGB1 and TGF-β1 (^§^*p* < 0.05). (**c**) Levels of collagen types I and III were detected by western blotting in HDF lysates treated with HMGB1 (100 ng) for 24 and 48 h. (**D**) The protein expression levels of collagen types I and III increased significantly after 24 h (**p < 0.01). (**e)** The protein expression level of α-SMA was assessed by western blotting in HDF cells treated with HMGB1 (50 and 100 ng). (**f**) Stimulation of HDFs with HMGB1 resulted in increased protein expression of α-SMA (**p < 0.01).

During its enhanced and prolonged release, HMGB1 acts as a profibrotic molecule by means of increased cell viability and collagen deposition of HDFs. We cultured HDFs in the presence of 50 ng and 100 ng of HMGB1 for the proliferation assay, and carried out a 3-(4,5-dimethylthiazol-2-yl)-2,5-diphenyltetrazolium bromide (MTT) cell assay. Significant time-dependent increase in proliferation activity in the HMGB1-treated group were noted (**p < 0.01; Fig. 2a). Therefore, HMGB1 has a significant positive effect on cell viability, and can activate fibroblasts in a manner that is similar to TGF-β1, a known fibrogenic cytokine.

Additionally, external stress such as exposure to LPS cans upregulate HMGB1 protein expression in HDFs. We evaluated whether HMGB1 can induce collagen synthesis and deposition. The effect of HMGB1 on collagen synthesis in normal HDFs was compared to TGF-β1 using quantitative reverse transcription polymerase chain reaction (qRT-PCR). Type I collagen mRNA expression was equivalently increased in HDFs treated with either HMGB1 (100 ng) or TGF-β1 (10 ng) (*p < 0.05, **p < 0.01; Fig. 2b). Furthermore, there was an additive increase in type I collagen mRNA expression after simultaneous treatment with HMGB1 and TGF-β1 (^§^*p* < 0.05; Fig. 2b), which was similar to KF. These results were confirmed by western blotting. The HDFs were treated with 100 ng/mL HMGB1 for 24 and 48 h. The protein expression levels of collagen types I and III significantly increased after 24 h (**p < 0.01; Fig. 2c and d). We also evaluated whether HMGB1 can differentiate HDF into myofibroblast. As shown in Fig. 2e and f, the expression level of α-smooth muscle actin (α-SMA) significantly increased in the presence of 50 ng and 100 ng of HMGB1.

### HMGB1 increased the expression of intracellular signaling such as the TGF-β1, Erk 1/2, Akt, and NF-κb cascades

**Figure 3:**
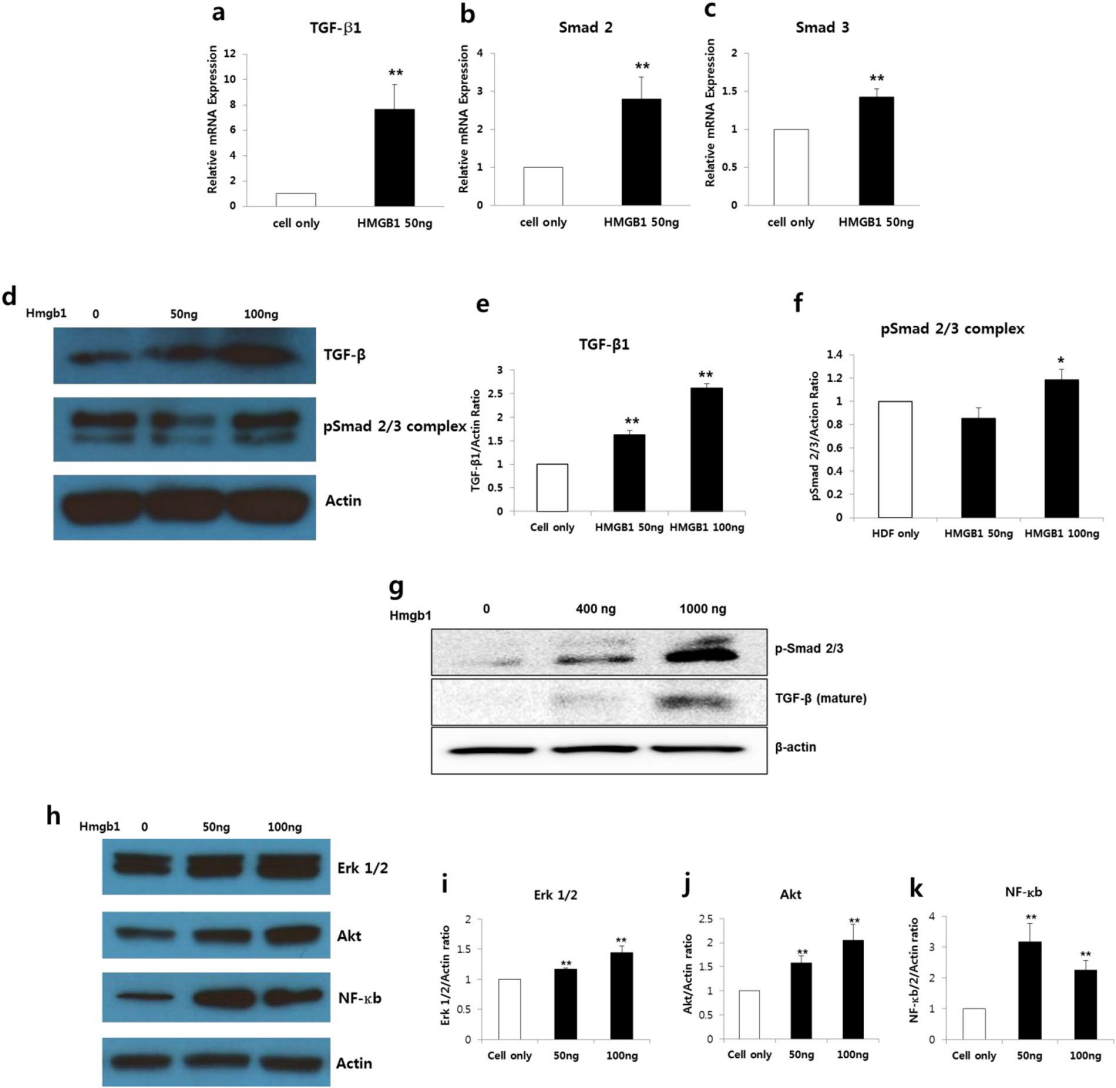
HMGB1 induced TGF-β1, Smads, Erk 1/2, Akt, and NF-κb in HDFs. (**a**–**c**) Effects of HMGB1 on TGF-β1 signaling pathway *in vitro*. Significant increases in the expression levels of TGF-β1, Smad 2, and Smad 3 mRNA were observed in the HMGB1 (50 ng)-treated HDFs (**p < 0.01). (**d**) Effects of HMGB1 on TGF-β and phosphor-Smad 2/3 complex protein expressions. (**e**,**f**) TGF-β and phosphor-Smad 2/3 complex protein levels were significantly increased in the HMGB1 (100 ng)-treated HDFs (**p < 0.01). (**g**) Western blotting analysis in normal dermal spheroids. Increased expression of TGF-β and phosphor-Smad 2/3 complex protein were observed in normal dermal spheroids treated with HMGB1 (400 ng and 1000 ng). (**h**) The levels of Erk 1/2, Akt, and NF-κb protein were detected by western blotting in HDFs lysates treated with HMGB1 (50 ng and 100 ng). (**i** to **k**) Erk 1/2, Akt, and NF-κb protein levels in HMGB1 (100 ng)-treated HDFs were significantly increased by 1.5-, 2.0-, and 2.3-fold, respectively, versus non-treated HDFs (**p < 0.01).

We next determined whether HMGB1-induced collagen synthesis is involved in the activation of TGF-β1/Smad signaling. Changes in the expression levels of profibrogenic TGF-β1 and Smad 2 and 3 following treatment with HMGB1 (50 ng) were investigated using real time RT-PCR and western blotting. TGF-β, Smad 2, and Smad 3 mRNA were significantly increased after HMGB1 treatment (**p < 0.01; Fig. 3a–c). Similar trends were observed at protein levels where HMGB1 treatment increased the expression level of TGF-β and phosphor-Smad 2/3 complex (**p < 0.01; Fig. 3d and f). Above effect of HMGB1 on TGF-β1 protein expression were confirmed using normal dermal spheroids with immunohistochemical analysis (Supplementary Figure S1) and western blotting. TGF-β1 and phospho-Smad 2/3 complex protein expressions increase when treated with HMGB1 (400 ng and 1000 ng) (Fig. 3g). In addition, the expression level of intracellular signaling molecules, such as Erk 1/2, Akt, and NF-κb, involved in collagen synthesis and myofibroblast differentiation were significantly elevated following HMGB1 treatment (**p < 0.01; Fig. 3h–k). Taken together, these results demonstrate that profibrogenic effect of HMGB1 is mediated by activation of TGF-β1/Smad signaling pathway.

### Effect of HMGB1 on MMP-1, MMP-2, and TIMP-1 mRNA expression

**Figure 4:**
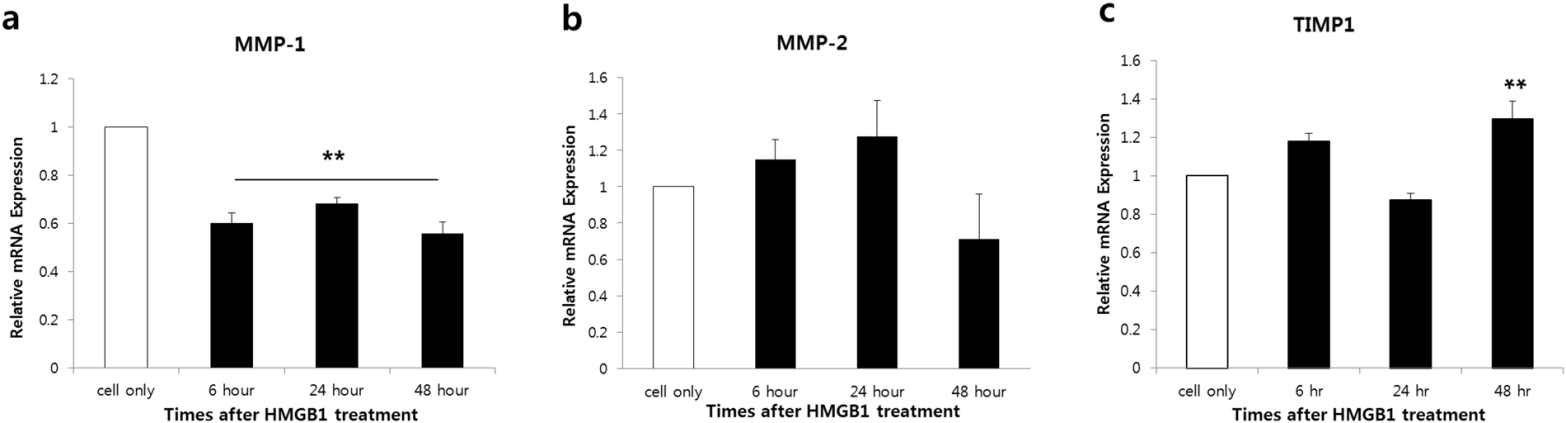
HMGB1 reduced MMP 1 and TIMP-1 mRNA expression. HDFs were treated with 50 ng/mL of HMGB1 for various times, and examined using real-time RT-PCR. (**a** and **c**) MMP-1 mRNA levels were significantly reduced, but TIMP1 mRNA level was increased in 48 hr after treatment with HMGB1 (**p < 0.01). (**b**) MMP-2 mRNA expression increased up to 24 h, but the increase was not statistically significant.

Dermal fibroblasts secrete matrix metalloproteinases (MMPs) to regulate extracellular matrix remodeling. Therefore, HMGB1 treatment can supposedly affect the expression of MMP-1, MMP-2, and TIMP-1 mRNA. As shown in Fig. 4, MMP-1 mRNA level was significantly reduced following treatment with HMGB1 (50 ng) (**p < 0.01; Fig. 4a), whereas TIMP1 mRNA level was significantly increased in 48 hr. post treatment with HMGB1 (**p < 0.01; Fig. 4c). In other word, MMP-1/TIMP1 mRNA ratio was significantly decreased following treatment with HMGB1 (50 ng) (**p < 0.01; Supplementary Figure S2). However, the expression of MMP-2 mRNA increased up to 24 h after treatment with HMGB1, although the increase was not statistically significant (Fig. 4b). These results suggest that HMGB1 regulates the expression of both MMP-1 and TIMP-1, which are major factors in collagen remodeling.

### Effect of HMGB1 on normal dermal spheroids

**Figure 5:**
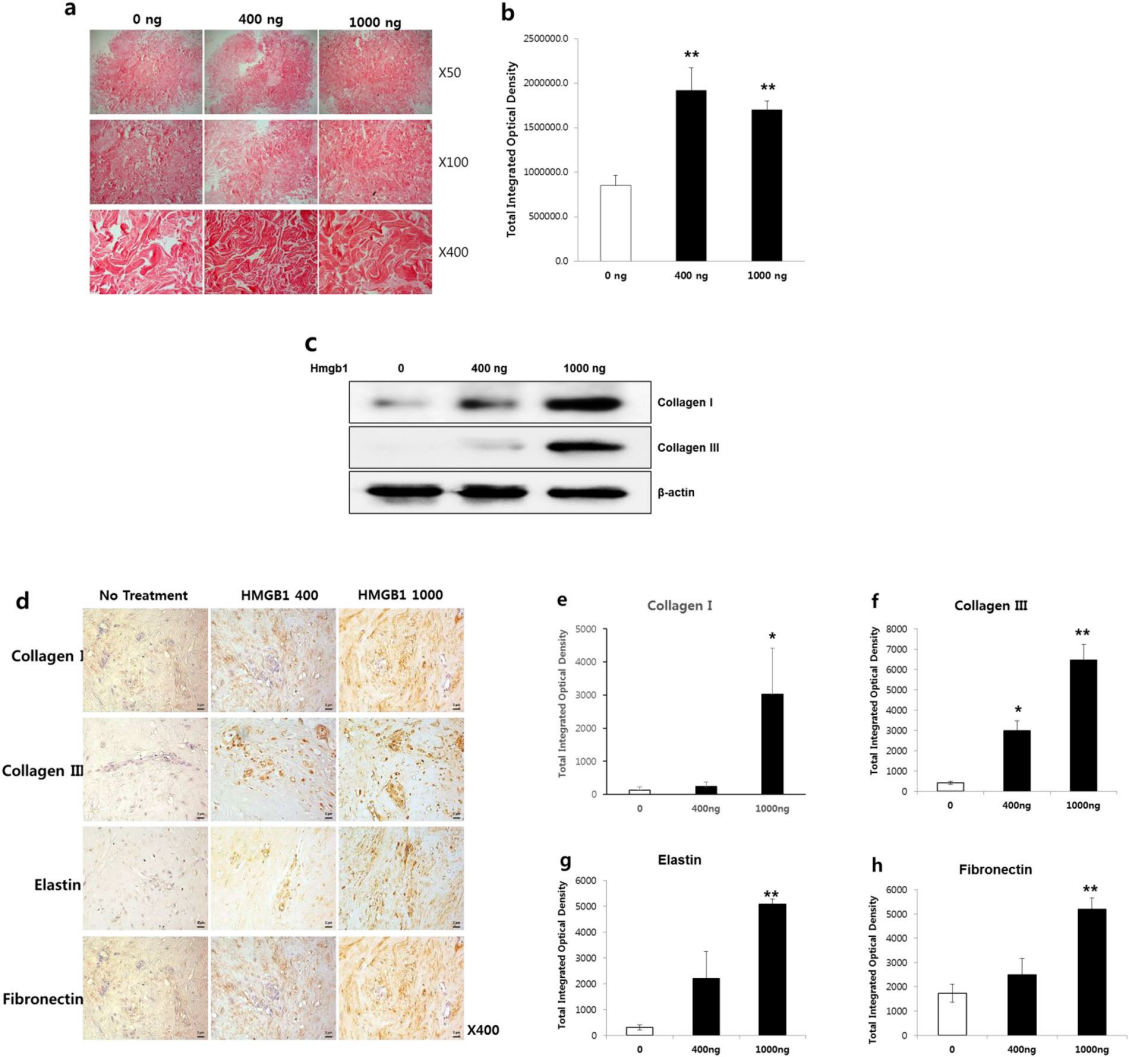
Effect of HMGB1 on normal dermal spheroids. (**a**) Picrosirius red staining of HMGB1-treated normal dermal spheroids. Collagen deposition increased. The HMGB1 dose dependency of dense and coarse collagen bundles was investigated. (**b**) Semi-quantitative analysis revealed that treatment with 400 and 1000 ng of HMGB1 significantly increased collagen deposition in normal dermal spheroids by 2.9- and 2-fold, respectively, versus non-treated dermal spheroids (**p < 0.01). (**c**) Western blotting analysis of HMGB1-treated normal dermal spheroids for the detection of type-I and -III collagen protein expression level (**d**) Immunohistochemical staining of HMGB1-treated normal spheroid sections for the detection of types I and III collagen, elastin, and fibronectin protein. Increased expression of ECM components (collagen types I and III, elastin, and fibronectin protein) was examined versus nontreated spheroids. Original magnification: 400×. (**e** to **h**) Semi-quantitative image analysis of ECM protein expression. The expression levels of type I collagen, type III collagen, elastin, and fibronectin increased significantly versus nontreated spheroids (*p < 0.05; **p < 0.01).

We investigated the effect of HMGB1 on normal skin tissue by *ex vivo* spheroid culture (n = 3). Various concentrations of HMGB1 were used to treat normal dermal spheroids. We analyzed the effect of HMGB1 on the expression of major extracellular matrix (ECM) components of normal dermal tissue. Picrosirius red staining revealed that collagen deposition increased in HMGB1-treated dermal spheroid sections, and formation of dense and coarse collagen bundles was dependent on the dose of HMGB1 (Fig. 5a). A semi-quantitative investigation using MetaMorph® image analysis software revealed that collagen deposition increased significantly by 2.9- and 2.0-fold in normal dermal spheroids treated with 400 and 1000 ng of HMGB1, respectively, versus untreated dermal spheroids (**p < 0.01; Fig. 5b). Similar results were obtained with western blotting where an increase of type-I and -III collagen protein levels were observed in dermal spheroids following treatment with HMGB1 (Fig. 5c). Image analysis of immunohistochemical staining also revealed that the expression levels of type I collagen, type III collagen, elastin, and fibronectin significantly increased by 22.4-, 15.5-, 16.4-, and 3-fold, respectively, in dermal spheroids treated with HMGB1 (1000 ng), versus non-treated spheroids (*p < 0.05, **p < 0.01; Fig. 5d–h), implying that HMGB1 plays a prominent role as a profibrogenic molecule during dermal fibrosis. Our results strongly suggest that HMGB1 could be an effective therapeutic target for treating abnormal dermal fibroses such as keloid and hypertrophic scars.

### Expression levels of HMGB1 and its receptors, such as RAGE and TLR4, increased in keloid tissue

**Figure 6:**
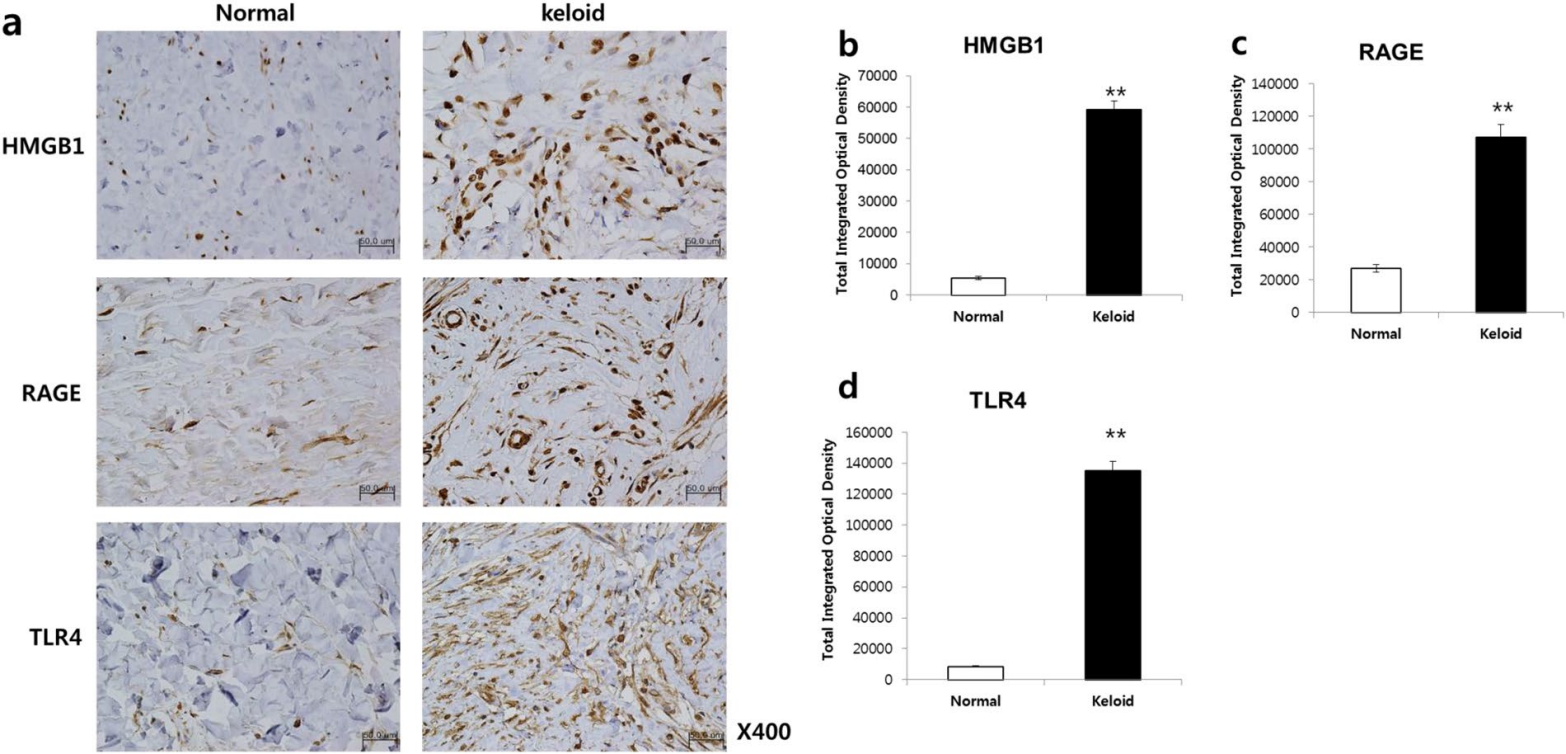
Immunohistochemical analysis of HMGB1 and its receptors (RAGE and TLR4) in human keloid tissues. (**a**) Higher expression levels of HMGB1 and its receptors (RAGE and TLR4) were observed in the region of the keloid tissues than in the adjacent normal tissue. Original magnification: 400×. (**b** to **d**) The comparison of HMGB1, RAGE, and TLR4 expression levels between keloid tissues and extra-lesional normal tissue (**p < 0.01).

We carried out immunohistochemical staining to investigate the expression patterns of HMGB1 and its receptors (RAGE and TLR4 proteins) in keloid tissue (n = 5). Hematoxylin and eosin (H&E) staining revealed that the keloid tissue had dense and excessive collagen deposits that extended over the clinical keloid margin into the extra-lesional dermal tissue (data not shown). Compared with extra-lesional normal tissue, markedly increased HMGB1, RAGE, and TLR4 immunoreactivity was noted in the central and peripheral keloid regions (Fig. 6a). Furthermore, we examined the excessive expression of HMGB1 in spindle-like cells such as fibroblasts. The increased expression of HMGB1 and its receptors was semi-quantitatively measured using MetaMorph® image analysis software (Fig. 6b–d). The expression levels of HMGB1, RAGE, and TLR4 in keloid tissues increased markedly by, 11-, 4-, and 15.8-fold, respectively, in comparison with extra-lesional normal tissue (**p < 0.01). These data suggest that HMGB1 and its effectors (RAGE and TLR4 proteins) are actively expressed in keloid tissues.

## Discussion

The inflammatory response is one of the major characteristics of keloid. However, the detailed mechanism has not been identified to date. Evidence suggests that signaling by HMGB1 or its receptors plays an essential role in mediating fibrotic diseases in the liver, kidneys, lung, and heart^13^. Therefore, we hypothesized that HMGB1, a well-known initiator of inflammation, might be associated with normal dermal fibrosis and keloid, as is TGF-β. In this study, we attempted to determine whether the pathological dermal fibrosis seen in keloid and hypertrophic scars resulted from excess HMGB1.

HMGB1 is abundant in all vertebrate nuclei, and serves as a nonhistone chromatin-binding protein for the stabilization of the chromatin structure and the modulation of gene transcription^17–19,27,28^. HMGB1 translocates from the nucleus into the cytosol, is released into the extracellular space during cell death or stress, and can act as a cytokine to mediate innate inflammation^13,29,30^. In the current study, we investigated the translocation and active release of HMGB1 from normal dermal fibroblasts during LPS stimulation, and the redistribution of nuclear HMGB1 into the cytoplasm of keloid fibroblasts. We also discovered an extracellular excess of HMGB1 and an increase in the levels of the HMGB1 receptors RAGE and TLR4 in the keloid tissue compared with the adjacent normal skin. Recent data have suggested that HMGB1 plays a role in wound healing by increasing the viability, proliferation, and migration of fibroblasts and keratinocytes^20,31–33^. However, there is scanty information on the relationship between skin fibrosis and HMGB1, especially in keloid. Therefore, we suggest that increased HMGB1 levels in keloid may account for the abnormal dermal fibrosis.

TGF-β is believed to play a key role in skin fibrosis and keloid pathophysiology^5,34–37^. Therefore, inhibiting TGF-β1-dependent signaling, using TGF-β1/TGF-β receptor-neutralizing antibodies, truncated receptors, antisense oligonucleotides, or Smad2-/Smad3-specific siRNAs decreases procollagen gene expression and inhibits fibrosis progression^2,6,34,38–40^. Our experimental results suggest that the role of HMGB1 is similar to those of TGF-β. Exogenous HMGB1 treatment can induce the proliferation of HDFs, production of several fibrogenesis related molecules (TIMP-1 mRNA, collagen, and α-SMA), and attenuation of ECM degrading MMP-1 mRNA expression level. Moreover, until now, the TGF-β/Smad, MARKs, Erk, and NF-κB pathways^7^ have been intensively investigated as the principle mechanisms underlining keloid scar formation^2,5,6,35,36^. Because our experiments revealed that TGF-β protein and several internal signaling molecules such as phosphor-Smad 2/3 complex, Erk 1/2, Akt, and NF-κb are overexpressed by HMGB1 in HDFs and normal tissues, modulating HMGB1 expression or its signaling is a potential therapeutic approach for preventing or inhibiting dermal fibrosis and keloid formation.

Several biological agents have been proposed for specifically inhibiting HMGB1, including antibodies, fragments of HMGB1 itself, and soluble receptors such as neutralizing anti-HMGB1 monoclonal antibodies, HMGB-A Box, and soluble RAGE. Ethyl pyruvate, glycyrrhizin, aspirin, quercetin, and methotrexate, which are introduced HMGB1 inhibitors, have also been studied^13,19,24,27,28,30^. Therefore, the clinical application of HMGB1 inhibitors for treating abnormal fibrosis has great potential. However, further research is required to identify safe candidates for the multitherapeutic control of the inflammation and HMGB1 biology involved in pathologic tissue fibrosis. In conclusion, the results of this study show that endogenous HMGB1 may be an essential profibrogenic molecule, and is a potential target for keloid tissue therapy.

## Materials and Methods

### Keloid tissues, human dermal fibroblasts, and normal abdominal tissues

Human keloid tissue samples were obtained after written informed consent and in accordance with a protocol approved by the Yonsei University College of Medicine Institutional Review Board (IRB No. 4-2015-0228). Keloid tissues from active-stage keloid patients (n = 12) and normal skin tissues obtained from abdomen, thigh, and back of healthy donors (n = 3) were obtained for fibroblast culture, histologic, and immunohistochemical analysis with excision (Supplementary Table 1). All experiments involving human tissues were performed in adherence to the Helsinki Guidelines. The primary human normal dermal fibroblasts (NFs) and keloid fibroblasts (KFs) were obtained from the ATCC (American Type Culture Collection, Manassas, VA, USA). Cells were cultured in Dulbecco’s modified Eagle’s medium (DMEM; Gibco, Grand Island, NY, USA) supplemented with heat-inactivated 10% fetal bovine serum and penicillin (100 U/mL), streptomycin (100 μg/mL), and actinomycin. The culture medium was changed at 2–3-day intervals. Both cells were used for experiment after 2~3 passage.

### Immunofluorescence assay

Cultured HDFs and KFs were washed twice with phosphate-buffered saline (PBS), fixed in 4% paraformaldehyde for 10 min at room temperature, and permeabilized by incubating for 15 min in 0.01% Tween® 20 in PBS. The samples were blocked with 5% bovine serum albumin and incubated with antibodies to HMGB1 (1:1000; Abcam, Cambridge, UK) overnight at 4 °C. The next day, the cells were washed with PBS and incubated with Alexa Fluor 488-conjugated goat anti-rabbit IgG (Invitrogen, Life Technologies, Grand Island, NY, USA) and Alexa Fluor 594-conjugated goat anti-mouse secondary antibody (Invitrogen) for 2 h at room temperature. The cells were mounted on slides using Vectashield® mounting medium with 4′, 6-diamidino-2-phenylindole (DAPI) (Vector Laboratories Inc., Burlingame, CA, USA) and viewed using a confocal microscope system (LSM700, Olympus Corp., Center Valley, PA, USA).

### Subcellular fractions for western blotting analysis

The nuclear/cytosolic fraction was obtained using a Nuclear/Cytosolic Fractionation kit (Cell Biolabs, San Diego, CA, USA) following the manufacturer’s instructions. To examine HMGB1 expression, HDFs and KFs were lysed in 50 mM Tris-HCl (pH 7.6), 1% Nonidet P-40 (NP-40), 150 mM NaCl, and 0.1 mM zinc acetate in the presence of protease inhibitors. Protein concentration was determined by the Lowry method (Bio-Rad, Hercules, CA, USA), and 30 g of each sample was separated by sodium dodecyl sulfate-polyacrylamide gel electrophoresis (SDS-PAGE). The proteins on the gel were electrotransferred to a polyvinylidene fluoride membrane, incubated with the primary mouse anti-HMGB1 monoclonal Ab (ab11354; Abcam, Cambridge, UK), rabbit anti-histone monoclonal Ab (#7631; Cell Signaling Technology, Beverly, MA, USA), or mouse anti-α-tubulin monoclonal Ab (#3873; Cell Signaling Technology, Beverly, MA, USA), and then incubated with the HRP (horseradish peroxidase)-conjugated secondary antibody (6160–05; Southern Bio Technology Associates, Inc., Birmingham, AL, USA). The expression patterns were revealed using an ECL detection kit (sc-2004; Santa Cruz Biotechnology, Santa Cruz, CA, USA). We carried out western blotting analysis to determine the effect of LPS (100 ng) and HMGB1 (100 ng) on the expression levels of HMGB1, collagen types I, collagen type III, α-SMA, TGF-β, and phospho-Smad 2/3 complex. We used primary mouse anti-HMGB1 monoclonal Ab (mAb; ab11354; Abcam, Cambridge, UK), mouse anti-collagen type-I mAb (Abcam), mouse anti-collagen type-III mAb (Sigma), rabbit anti-TGF-β1 mAb (Abcam), rabbit anti-phospho-Smad 2/3 mAb (Cell Signaling Technology), and actin mAb (mouse; Sigma-Aldrich, St. Louis, MO, USA).

### Methyl thiazolyl-diphenyl-tetrazolium bromide (MTT) cell viability assay

We carried out a 3-(4,5-dimethylthiazol-2-yl)-2,5-diphenyltetrazolium bromide (MTT) assay to evaluate cell proliferation (viability) and metabolic activity. We added HDFs (5 × 10^4^) to each well of a 24-well plate. The cells were incubated at 37 °C in fresh culture medium in an incubator with 5% CO_2_, the culture medium was removed, 200 μL of 0.5 mg/mL MTT solution (Boehringer, Mannheim, Germany) was added to each well, and the mixture was incubated at 37 °C for 3 h. The substrate medium was removed, 200 μL of dimethyl sulfoxide solution (Sigma) was added to each well, and the OD at 570 nm was read using an ELISA reader (Bio-Rad, Hercules, CA, USA).

### Quantitative real-time reverse transcriptase-polymerase chain reaction (qRT-PCR)

HDFs (2 × 10^5^ cells) were treated with HMGB1 (50 ng or 100 ng). At 3 days post-treatment, total RNA was prepared with TRIzol reagent (Gibco), and complementary DNA was prepared from 0.5 μg of total RNA by random priming using a first-strand cDNA synthesis kit (Promega), as described previously^41^.

### Primary normal dermal spheroid culture

Normal human dermal tissue samples from patients (n = 3, Supplementary Table 1) were obtained after written informed consent and in accordance with a protocol approved by the Yonsei University College of Medicine Institutional Review Board (IRB No. 4-2015-0228). All experiments involving human tissues were performed in adherence to the Helsinki Guidelines. Normal dermal spheroids were prepared by dissecting central dermal tissue into 2-mm diameter pieces with sterile 21-gauge needles. The explants were plated onto HydroCell® 24 multi-well plates (Nunc, Rochester, NY, USA), after which they were cultured for 4 h in Isocove’s modified Dulbecco’s medium (Gibco) supplemented with 5% FBS, 10 mM insulin (Sigma), and 1 mM hydrocortisone (Sigma). For histological analysis, HMGB1 protein (400 ng and 1000 ng) was added to the plates containing normal dermal spheroids, and incubated at 37 °C in a 5% CO_2_ incubator for 5 days. After cultivation of spheroids, we carried out western blotting analysis to determine the effect of HMGB1 (400 ng and 1000 ng) on the expression levels of collagen type I, collagen type III, TGF-β, and phospho-Smad 2/3 complex. We used primary mouse anti-collagen type-I (monoclonal Abcam, Cambridge, UK), mouse anti-collagen type-III mAb (Sigma), rabbit anti-TGF-β1 mAb (Abcam), rabbit anti-phosphor-Smad 2/3 mAb (Cell Signaling Technology), and actin antibodies were used (mouse monoclonal; Sigma-Aldrich, St. Louis, MO, USA).

### Histology and immunohistochemistry

Prepared normal dermal spheroids were then fixed with 10% formalin, paraffin-embedded, and cut into 5 μm-thick sections. Representative sections were stained with Picrosirius red, and then examined by light microscopy. For immunohistochemical staining, spheroid sections were incubated at 4 °C overnight with rabbit anti-TGF-β1 (Abcam), mouse anti-collagen type-I (Abcam), mouse anti-collagen type-III (Sigma), mouse anti-elastin (Sigma), or mouse anti-fibronectin (Santa Cruz Biotechnology) primary antibody, and then incubated at room temperature for 20 min with the Dako Envision™ Kit (Dako, Glostrup, Denmark) as a secondary antibody. Diaminobenzidine/hydrogen peroxidase (Dako) was used as the chromogen substrate. All slides were counterstained with Meyer’s hematoxylin. The expression levels of TGF-β1, collagen type I, collagen type III, elastin, and fibronectin were semi-quantitatively analyzed using MetaMorph® image analysis software (Universal Image Corp., Buckinghamshire, UK). The results are expressed as the mean optical density of six different digital images.

### Immunohistochemistry of HMGB1, RAGE, and TLR4 in keloid tissue

Formaldehyde-fixed tissues were transferred to a paraffin-embedded block, and 4 μm-thick sections were cut. After tissue deparaffination and rehydration, endogenous peroxidase activity was blocked by 10-min incubation at room temperature with absolute methanol containing 1% H_2_O_2_. The tissue sections were incubated with a primary antibody against rabbit anti-HMGB1 antibody (Abcam), rabbit anti-RAGE (Abcam), and monoclonal mouse anti-TLR4 (Imgenex, Midland, Canada) at 4 °C overnight. After incubation with secondary antibody (Super Sensitive™ Polymer-HRP IHC, Bio Genex) for 1 h at room temperature, the bound complexes were visualized by incubating the tissue sections with 0.05% diaminobenzidine and 0.003% H_2_O_2_. The sections were counterstained with Harris hematoxylin for nuclei, dehydrated, and mounted. The expression levels of HMGB1, TLR4, and RAGE were semi-quantitatively analyzed using MetaMorph® image analysis software (Molecular Devices, Sunnyvale, CA, USA). The results are expressed as the mean optical density (OD) for six different digital images per sample.

### Statistics analysis

The results are expressed as the mean ± standard deviation (SD). Data were analyzed by repeated one-way analysis of variance (ANOVA). Two sets of independent sample data were compared using a paired t-test; *p* < 0.05 was considered indicative of a statistically significant difference.

## Electronic supplementary material

Supplementary Information
