# Supplementary material for: Profibrogenic effect of high-mobility group box protein-1 in human dermal fibroblasts and its excess in keloid tissues
Source: Sci Rep. 2018 May 30;8:8434. doi: 10.1038/s41598-018-26501-6 (PMC5976629; doi:10.1038/s41598-018-26501-6)

Profibrogenic effect of high-mobility group box protein-1 in human dermal fibroblasts and its excess in keloid tissues

Won Jai Lee[1], Seung Yong Song[1], Hyun Roh[1], Hyo Min Ahn[2], Youjin Na[2], Jihee Kim[3], Ju Hee Lee[3§], Chae Ok Yun[2§]

[1]Institute for Human Tissue Restoration, Department of Plastic & Reconstructive Surgery, Yonsei University College of Medicine, Seoul, Korea

[2]Department of Bioengineering, College of Engineering, Hanyang University, Seoul, Korea

[3]Department of Dermatology and Cutaneous Biology Research Institute, Yonsei University College of Medicine, Seoul, Korea

Word count: 3457

Number of figures: 6, Number of tables: 0

Supplementary table : 1, Supplementary figure: 2

Financial Disclosure: The authors have no proprietary or commercial interest in any materials discussed in this article.

A short running head: Profibrogenic effect of HMGB1 in fibroblasts

[§]Corresponding author:

Chae Ok Yun, Ph.D.

Department of Bioengineering, College of Engineering,

Hanyang University, 222 Wangsimni-ro, Seongdong-gu, Seoul, Korea
E-mail: chaeok@hanyang.ac.kr Tel:+82-2-2220-0491. Fax:+82- 2-2220-1886

Ju Hee Lee, M. D., Ph.D.

Department of Dermatology and Cutaneous Biology Research Institute,

Yonsei University College of Medicine, 50 Yonsei-ro, Seodaemun-gu, Seoul, Korea

E-mail: juhee@yuhs.ac Tel: +82-2-2228-2080. Fax:+82-2-393-6947

Supplementary Table 1. Demographic information and descriptions of the keloids from the patients who participated in this study.

Case (No.)	Sex	Age (years)	Keloid Site	Others
K1	M	31	Chest	IHC
K2	F	33	Ear lobe	IHC
K3	F	11	Knee	IHC
K4	F	50	Anterior Chest	IHC
K5	F	5	Ankle	IHC
K6	F	18	Earlobe	WB
K7	F	17	Earlobe	WB, IHC
K8	M	53	Shoulder	WB, IHC<br>Immunofluorescence
K11	F	24	Earlobe	WB, IHC<br>Immunofluorescence
K12	F	33	Abdomen	WB, IHC<br>Immunofluorescence
N1	F	54	Abdomen	Spheroid, IHC
N2	F	46	Thigh	Spheroid, IHC
N3	M	54	Back	Spheroid, IHC<br>WB

Supplementary Figure S1. (**a**) Immunohistochemical analysis in normal dermal spheroids. (**b**) Increased expression of TGF-β1 protein was observed in normal dermal spheroids treated with HMGB1 (400 ng and 1000 ng) (**p < 0.01).

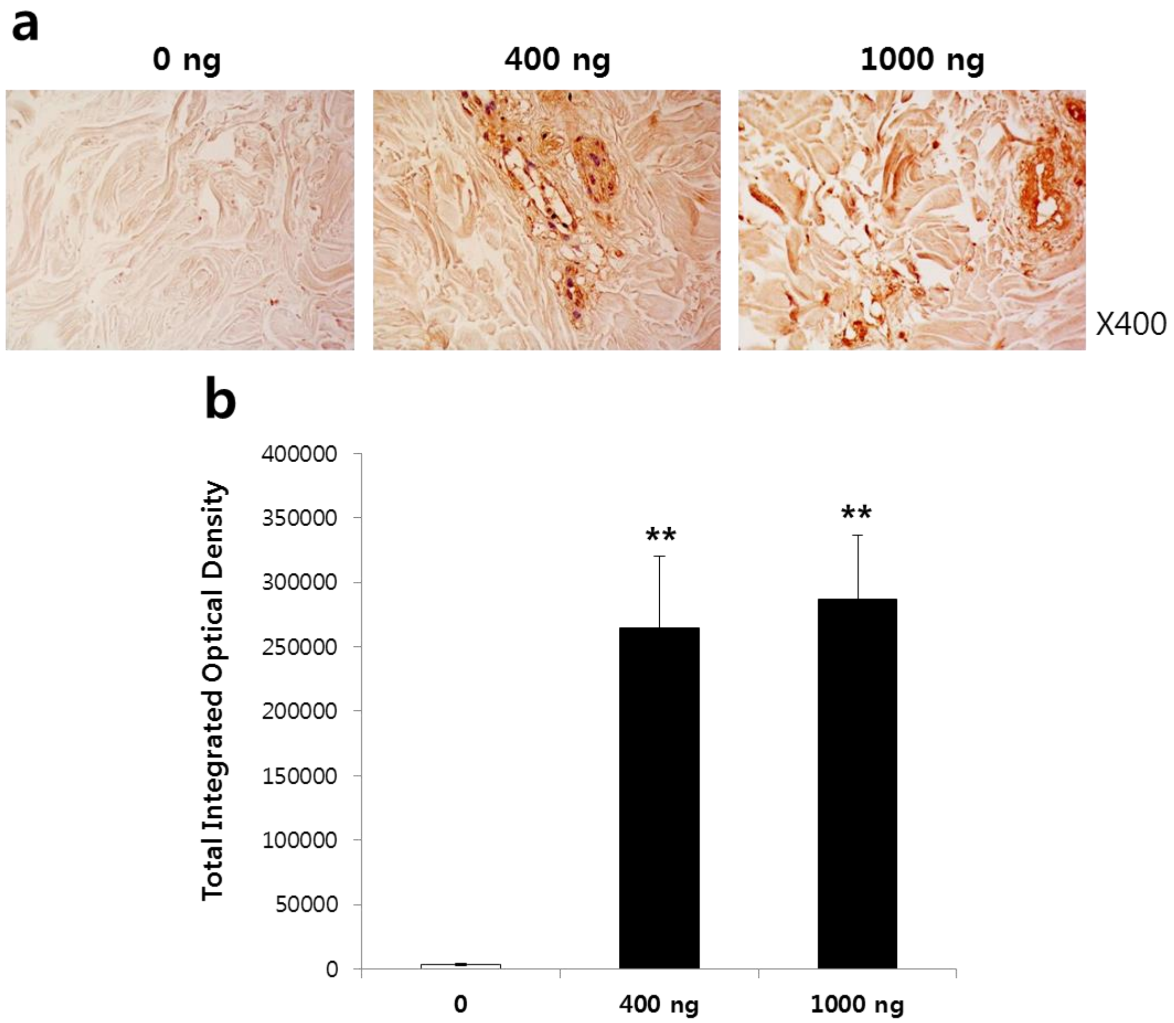

Supplementary Figure S2. MMP-1 mRNA/TIMP1 mRNA ratio. MMP-1/TIMP1 mRNA ratio was significantly decreased following treatment with HMGB1 (50 ng) (**$p < 0.01$)

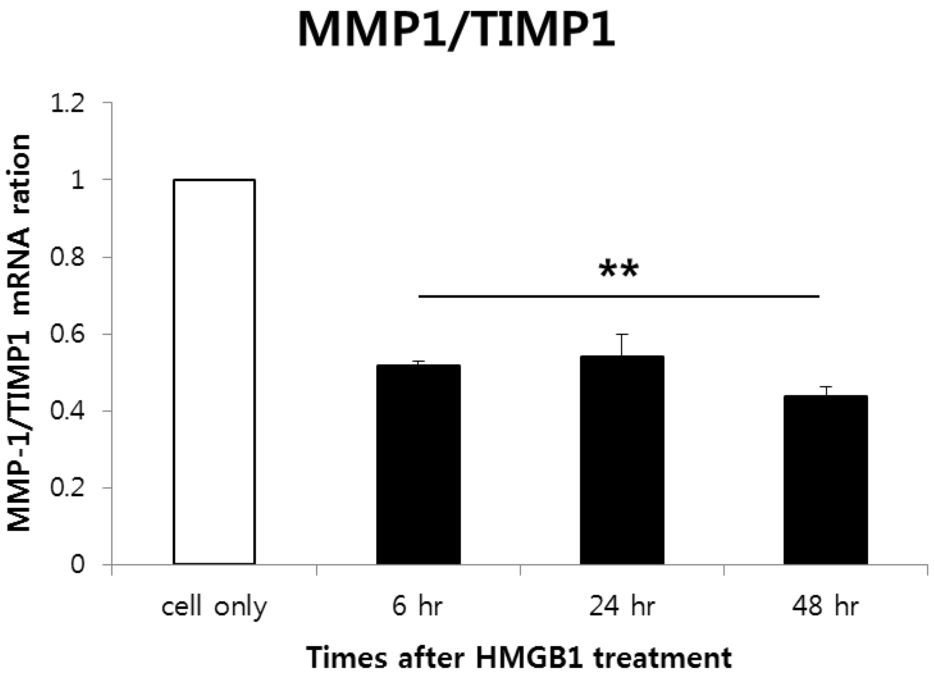

Supplement: Supplementary file 1 — Supplementary Information [file 41598_2018_26501_MOESM1_ESM.pdf]
